# Supplementary material for: Brain Death Determination: An Interprofessional Simulation to Determine Brain Death and Communicate with Families Focused on Neurology Residents
Source: MedEdPORTAL. 2020 Sep 25;16:10978. doi: 10.15766/mep_2374-8265.10978 (PMC7521065; doi:10.15766/mep_2374-8265.10978)
Supplement: Supplementary file 1 — Sample Schedule.docxCase 1.docxCase 1 Handout for Residents.docxCase 1 Handout for Family.docxCase 1 Handout for Nurse.docxCase 1 Handout for Chaplain.docxCase 1 Handout for Social Worker.docxCase 1 Head CT Scan.docxCase 2.docxCase 2 Handout for Residents.docxCase 2 Handout for Family.docxCase 2 Handout for Nurse.docxCase 2 Handout for Chaplain.docxCase 2 Handout for Social Worker.docxCase 2 Head CT Scan.docxCase 2 Angiography.docxCase 2 SPECT Scan.docxChecklist.docxPre and Postsimulation Survey.docx [file mep_2374-8265.10978-s001.zip › A. Sample Schedule.docx]

# Example of Schedule

Course duration: 4 hours

8:00-8:20 Introduction and Pretest

8:20-9:30 1st Simulation

- Exam (20 minutes)
- Huddle with social worker/chaplain/nurse (10 minutes)
- Family meeting (20 minutes)
- Debrief (20 minutes)

9:30-9:45 Break

9:45-11:00 2nd Simulation

- Prep meeting (20 minutes)
- Exam (20 minutes)
- Huddle with social worker/chaplain/nurse (15 minutes)
- Post meeting (20 minutes)

11:00-11:30 New England Organ Bank

11:30-12:00 Debrief and Posttest
