# Supplementary material for: Brain Death Determination: An Interprofessional Simulation to Determine Brain Death and Communicate with Families Focused on Neurology Residents
Source: MedEdPORTAL. 2020 Sep 25;16:10978. doi: 10.15766/mep_2374-8265.10978 (PMC7521065; doi:10.15766/mep_2374-8265.10978)
Supplement: Supplementary file 1 — Sample Schedule.docxCase 1.docxCase 1 Handout for Residents.docxCase 1 Handout for Family.docxCase 1 Handout for Nurse.docxCase 1 Handout for Chaplain.docxCase 1 Handout for Social Worker.docxCase 1 Head CT Scan.docxCase 2.docxCase 2 Handout for Residents.docxCase 2 Handout for Family.docxCase 2 Handout for Nurse.docxCase 2 Handout for Chaplain.docxCase 2 Handout for Social Worker.docxCase 2 Head CT Scan.docxCase 2 Angiography.docxCase 2 SPECT Scan.docxChecklist.docxPre and Postsimulation Survey.docx [file mep_2374-8265.10978-s001.zip › B. Case 1.docx]

| **Appendix B**  **SIMULATION CASE TITLE: Introductory Brain Death Determination**  **AUTHORS: Nicholas A. Morris, MD, Eli E. Zimmerman, MD**  **LEARNER AUDIENCE: Neurology Residents** | |
| --- | --- |
| **PATIENT NAME: Ms. Maguire**  **PATIENT AGE: 84 years old**  **CHIEF COMPLAINT: Unresponsive**  **PHYSICAL SETTING: Intensive Care Unit** | |
|  | |
| **Brief narrative description of case** | Residents perform a brain death determination in an unresponsive patient who has suffered a large intracerebral hemorrhage. They must recognize the indications for brain death determination and properly perform brain death testing. Next, they must deliver the diagnosis of brain death to the family of the deceased patient. |
| **Primary Learning Objectives** | Primary learning objectives include recognizing confounders to the brain death exam, demonstrating proficiency in the coma exam, performing and properly interpreting apnea testing, and delivering bad news to the family using the SPIKES methodology. |
| **Critical Actions** | - Asks to see neuroimaging to determine if it is compatible with brain death - Excludes confounders including metabolic disturbances, medication effects - Establishes hemodynamic stability (SBP ≥ 100 mm Hg) - Confirms absence of spontaneous respirations - Recognizes hypothermia and corrects - Pre-oxygenates the patient with 100% FiO2 - Checks for responsiveness to noxious stimuli at supraorbital nerve or temporomandibular joint as well as in all 4 extremities - Checks pupillary, corneal, oculocephalic, oculovestibular, gag, and cough reflexes - Confirms that patient is normocarbic prior to apnea testing (PaCO2 35-45 mm Hg) - Provides oxygen via suction catheter at level of carina at 6L/min - Disconnects ventilator - Uncovers chest and abdomen to observe for spontaneous effort - Checks ABG after 8-10 minutes of apnea - Declares and documents brain death at time of 2^nd^ ABG result - Huddles with interprofessional team to discuss approach to family meeting - Introduce and meet all parties - Ensure that setting is appropriate (chairs in circle, quiet, etc.) - Assess family’s understanding - Give warning shot - Use the term “death” - Provides date and time of death to family - Avoid medical jargon - Avoid terminology that perpetuates view of patient as alive (i.e. “life support,” “breathing on the ventilator,” etc.) - Offer silence - Respond to motions by attempting to name and understand them - Use ask-tell-ask approach - Give short-term plan with next steps - Offer space for final questions |
| **Learner Preparation or Prework** | - Cleveland Clinic’s online education in Death by Neurological Criteria (<https://www.cchs.net/onlinelearning/cometvs10/dncPortal/default.htm>) - Review a video tape of the coma exam - Review an article on giving bad news - Attend or watch a video of a lecture on brain death that reviewed our own hospital’s protocols and those of the New England Organ Bank - Attend a communication skills session. |

| Initial Presentation | | | |
| --- | --- | --- | --- |
| **Initial vital signs** | HR 70, BP 120/80, RR 14 (same as ventilator setting), O2sat 98%, T 92˚F | | |
| **Overall Setting and Appearance** | ICU room. Patient is comatose and connected to ventilator. An intensive care unit nurse and a respiratory therapist are present. | | |
| **Confederates (e.g., standardized participants) and their roles in the room at case start** | The confederates in the case are the patient’s two children, played by actors. | | |
| **HPI** | The following information is given to the resident:  Ms. Maguire is an 84-year-old woman with hypertension, hyperlipidemia, type 2 diabetes, breast cancer s/p chemo/XRT and GERD who is currently staying in a nursing home, after a fall at home resulting in a femur fracture. She was found to have a DVT while in the hospital and was started on full-dose anticoagulation with Enoxaparin.  At baseline, prior to her hospitalization and nursing home stay, she was suffering from pain from her osteoarthritis, and she had been having increasing difficulty managing her finances over the past 18 months. Her family (who lives out of state) was beginning to move towards moving her into an assisted-living facility.  Her husband passed away 2 years ago after a long battle with prostate cancer. During and after his illness, Ms. Maguire was clear in her wishes that she would never want to be maintained “on machines,” nor would she want to be dependent on assistance in feeding, bathing or dressing herself.  Last night, she was normal at dinnertime, and sleepier than usual when given her nightly medications. She usually complained about her Enoxaparin injections, but last night, she didn’t even flinch during the injection. This morning, she was unable to be roused for her morning pills and was breathing agonally. A code was called at the nursing home, where the patient was intubated without any medications. She was brought by ambulance to BWH for evaluation and received no medications en route. Neurosurgery was consulted and has declined to place an EVD.  Fast-forward 8 hours. She has already had one brain death exam, which revealed no brainstem reflexes or motor responses. | | |
| **Past Medical/Surgical History** | **Medications** | **Allergies** | **Family History** |
| Hypertension  Hyperlipidemia  Type 2 Diabetes Mellitus  Breast Cancer s/p lumpectomy, chemotherapy, and radiation  GERD  Deep Vein Thrombosis  Osteoarthritis | Enoxaparin  Lisinopril  Metformin  Simvastatin  Pantoprazole | No Known Drug Allergies | Non-contributory |
| **Physical Examination** | | | |
| **General** | Comatose | | |
| **HEENT** | Normocephalic, Atraumatic, no scleral icterus, normal conjunctivae, orally intubated | | |
| **Neck** | Supple | | |
| **Lungs** | Not breathing over the ventilator. Lungs clear to auscultation bilaterally | | |
| **Cardiovascular** | Regular rate and rhythm. No murmurs, rubs, gallops | | |
| **Abdomen** | Soft and nondistended | | |
| **Neurological** | No eye opening. No response to noxious stimuli above or below cervical spine. Pupils midsize and nonreactive. Absent corneal reflex. Absent oculocephalic and vestibulo-ocular reflex. Absent gag and cough. No motor responses to noxious stimulation of each limb. | | |
| **Skin** | No rashes | | |
| **GU** | Unremarkable | | |
| **Psychiatric** | Unable to test | | |

| Instructor Notes - Changes and CASE Branch Points  This simulation is the first of two. We intended to ease the residents into brain death determination and offer them a chance to practice the mechanics of the exam. As such, there are limited interventions or branch points. There are no consequences within the case for failure to perform critical actions. Instead, we discuss these deficiencies during the debriefing. | | |
| --- | --- | --- |
| **Intervention / Time point** | **Change in Case** | **Additional Information** |
| Hypothermia is recognized. | Bair Hugger is applied and temperature quickly climbs to 98˚F. |  |
| Baseline arterial blood gas is sent. | ABG returns: pH 7.40 / PaCO2 40 / PaO2 250  Apnea testing begins |  |
| Apnea testing is completed. | ABG returns: pH 7.16 / PaCO2 70 / PaO2 95  Patient is declared brain dead. |  |

**Ideal Scenario Flow**

The residents enter the intensive care unit room to find a comatose patient that is mechanically ventilated, an intensive care unit nurse, and a respiratory therapist. The residents review the case, including neuroimaging, and determine the cause of the coma to be known and irreversible. They rule out confounders to brain death determination through inquiry. They recognize hypothermia and correct it with surface warming. They perform a coma exam and document the absence of all cranial nerve reflexes and responsiveness. They correctly perform apnea testing and declare the patient dead at the time of the second arterial blood gas. They then huddle with the interprofessional team to discuss a plan for the family meeting. They meet with the family and divulge the diagnosis using best practices.

**Anticipated Management Mistakes**

1. Difficulty performing the apnea test: we found that residents are unsure about how to perform the apnea test. We included the respiratory therapist in the simulation to provide assistance and teaching during the simulation.
2. Difficulty stating the diagnosis to the family members: prior to the simulated family meeting, the interdisciplinary team met and if the resident did not volunteer his/her planned approach, the nurse and social worker would ask the resident how s/he planned to communicate the diagnosis.
