# Supplementary material for: Brain Death Determination: An Interprofessional Simulation to Determine Brain Death and Communicate with Families Focused on Neurology Residents
Source: MedEdPORTAL. 2020 Sep 25;16:10978. doi: 10.15766/mep_2374-8265.10978 (PMC7521065; doi:10.15766/mep_2374-8265.10978)
Supplement: Supplementary file 1 — Sample Schedule.docxCase 1.docxCase 1 Handout for Residents.docxCase 1 Handout for Family.docxCase 1 Handout for Nurse.docxCase 1 Handout for Chaplain.docxCase 1 Handout for Social Worker.docxCase 1 Head CT Scan.docxCase 2.docxCase 2 Handout for Residents.docxCase 2 Handout for Family.docxCase 2 Handout for Nurse.docxCase 2 Handout for Chaplain.docxCase 2 Handout for Social Worker.docxCase 2 Head CT Scan.docxCase 2 Angiography.docxCase 2 SPECT Scan.docxChecklist.docxPre and Postsimulation Survey.docx [file mep_2374-8265.10978-s001.zip › C. Case 1 Handout for Residents.docx]

# Case 1: Information for Residents

**Suggested timeline:**

Examination 20 minutes

Huddle with SW/chaplain/RN 10 minutes

Family meeting 20 minutes

Debrief 20 minutes

**Background information:**

Ms. Maguire is an 84-year-old woman with hypertension, hyperlipidemia, type 2 diabetes, breast cancer s/p chemo/XRT and GERD who is currently staying in a nursing home, after a fall at home resulting in a femur fracture. She was found to have a DVT while in the hospital and was started on full-dose anticoagulation with Lovenox.

At baseline, prior to her hospitalization and nursing home stay, she was suffering from pain from her osteoarthritis, and she had been having increasing difficulty managing her finances over the past 18 months. Her family (who lives out of state) was beginning to move towards moving her into an assisted-living facility.

Her husband passed away 2 years ago after a long battle with prostate cancer. During and after his illness, Ms. Maguire was clear in her wishes that she would never want to be maintained “on machines,” nor would she want to be dependent on assistance in feeding, bathing or dressing herself.

Last night, she was normal at dinnertime, and sleepier than usual when given her nightly medications. She usually complained about her Lovenox injections, but last night, she didn’t even flinch during the injection. This morning, she was unable to be roused for her morning pills and was breathing agonally. A code was called at the nursing home, where the patient was intubated without any medications. She was brought by ambulance to the hospital for evaluation and received no medications en route. Neurosurgery was consulted and has declined to place an EVD.

Fast-forward 8 hours. She has already had one brain death exam, which revealed no brainstem reflexes or motor responses.

**Situation:**

You have already had an initial meeting with the family, where you discussed what with the knowledge of what the first exam showed, as you performed this exam with the attending on rounds. You have already told them that you will be repeating this exam. Your job in this case is to perform the brain death testing, huddle with the interdisciplinary team, then discuss the results with the family.

**General guidelines for family meeting:**

1. Introduce and meet all parties.
2. Ensure that the setting is appropriate (chairs in a circle, quiet room, pager turned off).
3. Ask for family’s understanding of the situation.
4. Give warning shot.
5. Succinctly summarize scenario and its implications.
6. Use the term “death.” Avoid vague language.
7. Offer silence.
8. Respond to family’s emotions and try to clarify and understand them.
9. Use ask-tell-ask approach.
10. Give short-term plan with next steps.
11. Offer space for final questions.
