# Supplementary material for: Brain Death Determination: An Interprofessional Simulation to Determine Brain Death and Communicate with Families Focused on Neurology Residents
Source: MedEdPORTAL. 2020 Sep 25;16:10978. doi: 10.15766/mep_2374-8265.10978 (PMC7521065; doi:10.15766/mep_2374-8265.10978)
Supplement: Supplementary file 1 — Sample Schedule.docxCase 1.docxCase 1 Handout for Residents.docxCase 1 Handout for Family.docxCase 1 Handout for Nurse.docxCase 1 Handout for Chaplain.docxCase 1 Handout for Social Worker.docxCase 1 Head CT Scan.docxCase 2.docxCase 2 Handout for Residents.docxCase 2 Handout for Family.docxCase 2 Handout for Nurse.docxCase 2 Handout for Chaplain.docxCase 2 Handout for Social Worker.docxCase 2 Head CT Scan.docxCase 2 Angiography.docxCase 2 SPECT Scan.docxChecklist.docxPre and Postsimulation Survey.docx [file mep_2374-8265.10978-s001.zip › D. Case 1 Handout for Family.docx]

## Case 1: Information for Family

Your mom, Linda, is hospitalized after being found unresponsive at the nursing home. A breathing tube was placed prior to coming to the hospital.

You have already had long conversations with her about her wishes in this type of situation, particularly when your father (Peter) was sick with prostate cancer. She was clear in her wishes not to be kept alive “on machines” or to be dependent on others for feeding, bathing or dressing herself. She has been staying in a nursing home after she had a fall down some stairs leaving bingo a few weeks ago, but you and your siblings have already been talking about moving her into an assisted-living facility closer to the family, as she has had some troubles balancing her checkbook and getting around safely. She and your father had a home in Boston, and she has been adamant about wanting to stay there; you and your siblings are ready to move her to New Jersey, where everyone else lives.

The doctors in the ER called you last night after your mother was brought into the hospital and told you what had happened. The neurology doctors in the ICU have told you what is going on, and that her brain is not working well. They explained that they were going to do some exams on her to see if she was alive, and that the first one showed that her brain was not functioning.

You have just arrived at the hospital after driving all night from New Jersey.

**Suggested questions to ask:**

What is going on?

Is she alive?

Could we have prevented this?

What are all the monitors saying?

Is she breathing?

Is she in pain?

What would you do, if this was your mom?
