# Supplementary material for: Brain Death Determination: An Interprofessional Simulation to Determine Brain Death and Communicate with Families Focused on Neurology Residents
Source: MedEdPORTAL. 2020 Sep 25;16:10978. doi: 10.15766/mep_2374-8265.10978 (PMC7521065; doi:10.15766/mep_2374-8265.10978)
Supplement: Supplementary file 1 — Sample Schedule.docxCase 1.docxCase 1 Handout for Residents.docxCase 1 Handout for Family.docxCase 1 Handout for Nurse.docxCase 1 Handout for Chaplain.docxCase 1 Handout for Social Worker.docxCase 1 Head CT Scan.docxCase 2.docxCase 2 Handout for Residents.docxCase 2 Handout for Family.docxCase 2 Handout for Nurse.docxCase 2 Handout for Chaplain.docxCase 2 Handout for Social Worker.docxCase 2 Head CT Scan.docxCase 2 Angiography.docxCase 2 SPECT Scan.docxChecklist.docxPre and Postsimulation Survey.docx [file mep_2374-8265.10978-s001.zip › E. Case 1 Handout for Nurse.docx]

## Case 1: Information for RN

Ms. Maguire is an 84-year-old woman with hypertension, hyperlipidemia, type 2 diabetes, breast cancer s/p chemo/XRT and GERD who is currently staying in a nursing home, after a fall at home resulting in a femur fracture. She was found to have a DVT while in the hospital and was started on full-dose anticoagulation with Lovenox.

At baseline, prior to her hospitalization and nursing home stay, she was suffering from pain from her osteoarthritis, and she had been having increasing difficulty managing her finances over the past 18 months. Her family (who lives out of state) was beginning to move towards moving her into an assisted-living facility.

Her husband passed away 2 years ago after a long battle with prostate cancer. During and after his illness, Ms. Maguire was clear in her wishes that she would never want to be maintained “on machines,” nor would she want to be dependent on assistance in feeding, bathing or dressing herself.

Last night, she was normal at dinnertime, and sleepier than usual when given her nightly medications. She usually complained about her Lovenox injections, but last night, she didn’t even flinch during the injection. This morning, she was unable to be roused for her morning pills and was breathing agonally. A code was called at the nursing home, where the patient was intubated without any medications. She was brought by ambulance to the hospital for evaluation and received no medications en route. Neurosurgery was consulted and has declined to place an EVD.

Fast-forward 8 hours. She has already had one brain death exam, which revealed no brainstem reflexes or motor responses.

You were the nurse taking care of Ms. Maguire when she came into the hospital yesterday afternoon (working 3-11pm), and had some conversations with her daughter and son-in-law over the phone last night before you left. You have told them that she is very sick, and they are pretty clear in stating what her wishes were (she is DNR/DNI, though she has already been intubated). The team has performed one brain death exam already, and she had fixed and dilated pupils, no cough, no gag, and she was not overbreathing the vent. No movement in her arms or legs with pinching.

Her family has just arrived from out of state.
