# Supplementary material for: Brain Death Determination: An Interprofessional Simulation to Determine Brain Death and Communicate with Families Focused on Neurology Residents
Source: MedEdPORTAL. 2020 Sep 25;16:10978. doi: 10.15766/mep_2374-8265.10978 (PMC7521065; doi:10.15766/mep_2374-8265.10978)
Supplement: Supplementary file 1 — Sample Schedule.docxCase 1.docxCase 1 Handout for Residents.docxCase 1 Handout for Family.docxCase 1 Handout for Nurse.docxCase 1 Handout for Chaplain.docxCase 1 Handout for Social Worker.docxCase 1 Head CT Scan.docxCase 2.docxCase 2 Handout for Residents.docxCase 2 Handout for Family.docxCase 2 Handout for Nurse.docxCase 2 Handout for Chaplain.docxCase 2 Handout for Social Worker.docxCase 2 Head CT Scan.docxCase 2 Angiography.docxCase 2 SPECT Scan.docxChecklist.docxPre and Postsimulation Survey.docx [file mep_2374-8265.10978-s001.zip › F. Case 1 Handout for Chaplain.docx]

## Case 1: Information for Chaplain

You have been called to see the family of Linda Maguire, who is an 84-year-old woman who has been admitted to the ICU after suffering a devastating and unsurvivable brain hemorrhage. You actually know her from when she was in the hospital last month for a blood clot, and you know that she is a devout Catholic, and that until this recent illness, she went to church every Sunday, taught in the Sunday School, and worked with the church’s volunteer efforts.

You have never met her family, but Ms. Maguire spoke very highly about her children, and how close they are.
