# Supplementary material for: Brain Death Determination: An Interprofessional Simulation to Determine Brain Death and Communicate with Families Focused on Neurology Residents
Source: MedEdPORTAL. 2020 Sep 25;16:10978. doi: 10.15766/mep_2374-8265.10978 (PMC7521065; doi:10.15766/mep_2374-8265.10978)
Supplement: Supplementary file 1 — Sample Schedule.docxCase 1.docxCase 1 Handout for Residents.docxCase 1 Handout for Family.docxCase 1 Handout for Nurse.docxCase 1 Handout for Chaplain.docxCase 1 Handout for Social Worker.docxCase 1 Head CT Scan.docxCase 2.docxCase 2 Handout for Residents.docxCase 2 Handout for Family.docxCase 2 Handout for Nurse.docxCase 2 Handout for Chaplain.docxCase 2 Handout for Social Worker.docxCase 2 Head CT Scan.docxCase 2 Angiography.docxCase 2 SPECT Scan.docxChecklist.docxPre and Postsimulation Survey.docx [file mep_2374-8265.10978-s001.zip › I. Case 2.docx]

| **Appendix I**  **SIMULATION CASE TITLE: Advanced Brain Death Determination**  **AUTHORS: Nicholas A. Morris, MD, Eli E. Zimmerman, MD**  **LEARNER AUDIENCE: Neurology Residents** | |
| --- | --- |
| **PATIENT NAME: Mr. O’Reilly**  **PATIENT AGE: 58 years-old**  **CHIEF COMPLAINT: Unresponsive**  **PHYSICAL SETTING: Intensive Care Unit** | |
|  | |
| **Brief narrative description of case** | Residents meet with family to introduce the concept of brain death and prepare them for testing. Residents then perform a brain death determination in an unresponsive patient who has suffered a traumatic cardiac arrest and has completed therapeutic hypothermia. Due to traumatic injuries, the residents cannot complete a neurological exam cannot be completed and an ancillary test is indicated. In addition, the residents must abort apnea testing due to hypoxia. Following an ancillary test that confirms brain death, the residents meet again with the family. The interjections of a newly arrived sibling who challenges the concept of brain death and invokes religious objections complicate the second meeting. |
| **Primary Learning Objectives** | Primary learning objectives include recognizing confounders to the brain death exam, appropriate utilization of ancillary testing, and supporting family that is resistant to the diagnosis of brain death. |
| **Critical Actions** | - Introduce and meet all parties - Ensure that setting is appropriate (chairs in circle, quiet, etc.) - Assess family’s understanding - Avoid medical jargon - Use ask-tell-ask approach - Give short-term plan with next steps - Asks to see neuroimaging to determine if it is compatible with brain death - Excludes confounders including metabolic disturbances, medication effects - Establishes hemodynamic stability (SBP ≥ 100 mm Hg) - Confirms absence of spontaneous respirations - Pre-oxygenates the patient with 100% FiO2 - Checks for responsiveness to noxious stimuli at supraorbital nerve or temporomandibular joint as well as in all 4 extremities - Checks pupillary, corneal, oculocephalic, oculovestibular, gag, and cough reflexes - Notes inability to check R pupillary, R corneal, and oculovestibular reflexes. - Confirms that patient is normocarbic prior to apnea testing (PaCO2 35-45 mm Hg) - Provides oxygen via suction catheter at level of carina at 6L/min - Disconnects ventilator - Uncovers chest and abdomen to observe for spontaneous effort - Aborts apnea testing after > 30 s of O2sat < 85% - Orders appropriate ancillary test (digital subtraction angiography, SPECT scan) - Interprets ancillary test as consistent with brain death and documents brain death at time of ancillary test. - Huddles with interprofessional team to discuss approach to family meeting - Introduce and meet all parties (again) - Re-assess family’s understanding - Give warning shot - Avoid medical jargon - Use the word “death” - Avoid terminology that perpetuates view of patient as alive (i.e. “life support”, “breathing on the ventilator”, etc.) - Provides date and time of death to family - Offer silence - Respond to emotions by attempting to name and understand them - Incorporates chaplain into response to sibling’s religious objections - Uses visual aids (CT, DSA, SPECT) appropriately - Uses “active listening” techniques: parrots and paraphrases - Uses repeated and/or multiple explanations to achieve comprehension by all family members - If appropriate, offer observation of neurological exam - Give short-term plan with next steps - Offer space for final questions |
| **Learner Preparation or Prework** | - Cleveland Clinic’s online education in Death by Neurological Criteria (<https://www.cchs.net/onlinelearning/cometvs10/dncPortal/default.htm>) - Review a video tape of the coma exam - Review an article on giving bad news - Attend or watch a video of a lecture on brain death that reviewed our own hospital’s protocols and those of the New England Organ Bank - Attend a communication skills session. - Complete Brain Death Determination Case 1. |

| Initial Presentation | | | |
| --- | --- | --- | --- |
| **Initial vital signs** | HR 70, BP 120/80, RR 14 (same as ventilator setting), O2sat 98%, T 92˚F | | |
| **Overall Setting and Appearance** | Two main settings–one family meeting room and one intensive care unit room. In the family meeting room, the residents are greeted by the patient’s wife and 1 or 2 adult children in the first meeting. In the follow-up meeting they are also joined by the patient’s sibling. In the intensive care room, the patient is comatose and connected to ventilator. An intensive care unit nurse and a respiratory therapist are in the intensive care unit room. | | |
| **Confederates (e.g., standardized participants) and their roles in the room at case start** | The confederates in the case are the patient’s wife, child (or children), and sibling, all played by actors. | | |
| **HPI** | Mr. O’Reilly is a 58-year-old man with no prior medical problems who was brought to the ED 8 days ago after falling off a 3-story roof onto the sidewalk. He had been complaining of chest pain that morning, though he ascribed it to a recent upper respiratory infection. After falling onto the sidewalk, a nurse who was out for a jog checked on him, and when she could not find a pulse, she started CPR. When EMS arrived 20 minutes later, he had not regained spontaneous circulation. Initial rhythm by EMS was non-shockable, and CPR continued for the 25 minutes until he arrived in the ED. There, he regained spontaneous circulation. A trauma CT revealed a basilar skull fracture, fractures of ribs 2-10 on the right, a right nondisplaced clavicle fracture, comminuted right humerus fractures, a right femoral neck fracture and a right tibial plateau fracture. A non-contrast head CT did not show intracranial hemorrhage. He was begun on a therapeutic hypothermia protocol, and the neurology consult service was called. When he was fully rewarmed, his exam remained poor, despite having been off sedation for 36 hours. He has remained off sedation for 4 days with no change in his exam. | | |
| **Past Medical/Surgical History** | **Medications** | **Allergies** | **Family History** |
| None | None | No Known Drug Allergies | Non-contributory |
| **Physical Examination** | | | |
| **General** | Comatose | | |
| **HEENT** | Right periorbital edema and ecchymosis such that the eyelid cannot be opened. R hemotympanum with clotted blood obstructing the canal, orally intubated | | |
| **Neck** | Miami-J Collar in place | | |
| **Lungs** | Not breathing over the ventilator. Lungs clear to auscultation bilaterally | | |
| **Cardiovascular** | Regular rate and rhythm. No murmurs, rubs, gallops | | |
| **Abdomen** | Soft and non-distended | | |
| **Neurological** | No eye opening. No response to noxious stimuli above or below cervical spine. L pupils midsize and nonreactive but R pupil unable to be seen due to periorbital edema. Absent corneal reflex on L. Unable to check L corneal reflex. Absent oculocephalic and vestibulo-ocular reflex. Absent gag and cough. No motor responses to noxious stimulation of each limb. | | |
| **Skin** | No rashes | | |
| **GU** | Unremarkable | | |
| **Musculoskeletal** | Surgical immobilizers on RUE and RLE | | |

| Instructor Notes - Changes and CASE Branch Points  This simulation is the second of two. This case is more challenging. Residents must recognize that the coma exam and apnea testing cannot be completed and order appropriate ancillary testing. Residents must There are no consequences within the case for failure to perform critical actions. Instead, we discuss these deficiencies during the debriefing. | | |
| --- | --- | --- |
| **Intervention / Time point** | **Change in Case** | **Additional Information** |
| Recognize traumatic injuries that limit coma exam. | Order appropriate ancillary testing. |  |
| Baseline arterial blood gas is sent. | ABG returns: pH 7.40 / PaCO2 40 / PaO2 201  Apnea testing begins |  |
| Patient’s oxygen levels decline to < 85% for > 30s. | Consider repeating procedure with T-piece, CPAP 10 cm H2O, and 100% O2 12 L/min. Order appropriate ancillary testing. |  |
| Apnea testing repeated with T-Piece, CPAP 10 cm H2O, and 100% O2 12 L/min. | Patient’s oxygen levels decline to < 85% for > 30s. Ancillary testing ordered |  |

**Ideal Scenario Flow**

The residents meet with the patient’s wife and adult child (or children) to prepare them for the brain death determination. They then move to the patient’s intensive care unit room where they are joined by the patient’s nurse and the respiratory therapist. They successfully complete all testable components of the brain death exam, while recognizing that they will be unable to check the right pupillary, right corneal, and vestibulo-ocular reflexes. They do attempt to perform apnea testing, and appropriately abort the apnea test for hypoxia. They then order ancillary testing which they correctly interpret as consistent with brain death. The residents then return to meet with the family following a brief interprofessional huddle. Joining the already introduced family members is a sibling of the patient who “just arrived from out-of-town.” The residents must adeptly lead the family meeting, carefully re-explaining things to the newly arrived sibling and incorporating the interprofessional team to appropriately handle the sibling’s objections to the diagnosis of brain death.

**Anticipated Management Mistakes**

1. Uncertainty about which ancillary test to use: we had results of all possible ancillary tests available and provided teaching after the simulation about how to best use the different ancillary tests.
2. Difficulty handling sibling who “just arrived from out of town” with appeals to religion and miracles: we embedded the chaplain and social worker within the difficult family meeting to assist.
