# Supplementary material for: Brain Death Determination: An Interprofessional Simulation to Determine Brain Death and Communicate with Families Focused on Neurology Residents
Source: MedEdPORTAL. 2020 Sep 25;16:10978. doi: 10.15766/mep_2374-8265.10978 (PMC7521065; doi:10.15766/mep_2374-8265.10978)
Supplement: Supplementary file 1 — Sample Schedule.docxCase 1.docxCase 1 Handout for Residents.docxCase 1 Handout for Family.docxCase 1 Handout for Nurse.docxCase 1 Handout for Chaplain.docxCase 1 Handout for Social Worker.docxCase 1 Head CT Scan.docxCase 2.docxCase 2 Handout for Residents.docxCase 2 Handout for Family.docxCase 2 Handout for Nurse.docxCase 2 Handout for Chaplain.docxCase 2 Handout for Social Worker.docxCase 2 Head CT Scan.docxCase 2 Angiography.docxCase 2 SPECT Scan.docxChecklist.docxPre and Postsimulation Survey.docx [file mep_2374-8265.10978-s001.zip › J. Case 2 Handout for Residents.docx]

# Case 2: Information for Residents

**Suggested timeline:**

Huddle with SW/chaplain/RN 5-10 minutes

Family discussion 20 minutes

Examination 15 minutes

Huddle with SW/chaplain/RN 5-10 minutes

Family meeting 20 minutes

**Background information:**

Mr. O’Reilly is a 58-year-old man with no prior medical problems who was brought to the hosptial ED 8 days ago after falling off a 3-story roof onto the sidewalk. He had been complaining of chest pain that morning, though he ascribed it to a recent upper respiratory infection. After falling onto the sidewalk, a nurse who was out for a jog checked on him, and when she could not find a pulse, she started CPR. When EMS arrived 20 minutes later, he had not regained spontaneous circulation. Initial rhythm by EMS was non-shockable, and CPR continued for the 25 minutes until he arrived in the ED. There, he regained spontaneous circulation. A trauma CT revealed a basilar skull fracture, fractures of ribs 2-10 on the right, a right nondisplaced clavicle fracture, comminuted right humerus fractures, a right femoral neck fracture and a right tibial plateau fracture. A non-contrast head CT did not show intracranial hemorrhage. He was begun on a therapeutic hypothermia protocol, and the neurology consult service was called. When he was fully rewarmed, his exam remained poor, despite having been off sedation for 36 hours. He has remained off sedation for 4 days with no change in his exam.

**Situation:**

You have had multiple discussions with his wife and adult children, who have been present in the hospital throughout his stay. You have been keeping them updated daily about his neurologic status. After a meeting with the family and all consulting teams yesterday (SICU, Neurology, Cardiology), all have agreed that there are no further interventions to be done. The neurology team has followed the patient’s exam serially and today’s exam revealed absent brainstem and motor reflexes. You and the team are preparing for brain death testing. Your job is to:

1. huddle with the interdisciplinary team,
2. have a meeting with the family to prepare them for what you will be doing,
3. examine the patient,
4. re-huddle with the team,
5. and finally discuss your findings and the next steps with the family.

**General guidelines for “preparatory” family meeting:**

1. Introduce and meet all parties.
2. Ensure that the setting is appropriate (chairs in a circle, quiet room, pager turned off).
3. Ask for family’s understanding of the situation.
4. Give warning shot.
5. Succinctly summarize scenario and its implications.
6. Use the term “death.” Avoid vague language.
7. Offer silence.
8. Respond to family’s emotions and try to clarify and understand them.
9. Use ask-tell-ask approach.
10. Give short-term plan with next steps.
11. Offer space for final questions.

**General guidelines for post-exam family meeting:**

1. Avoids suggestions of “hope” for recovery and possibility of a “miracle.”
2. Encourages families to ask questions.
3. Provides the date and/or time of death.
4. Uses simple words and phrases to explain clinical terms.
5. Uses the word “dead.”
6. Uses visual aids (CT, X-ray, SPECT, angio) appropriately.
7. Explains findings and/or components of brain protocol, neurologic exams, apnea test, or confirmatory tests for brain death.
8. Checks for comprehension of information by family member(s).
9. Uses “active listening”: parroting and paraphrasing.
10. Uses repeated and/or multiple explanations to achieve comprehension by family member(s).
11. If appropriate, offers observation of neurologic examination.
12. Explains next decision-making steps without mention of organ donation.
