# Supplementary material for: Brain Death Determination: An Interprofessional Simulation to Determine Brain Death and Communicate with Families Focused on Neurology Residents
Source: MedEdPORTAL. 2020 Sep 25;16:10978. doi: 10.15766/mep_2374-8265.10978 (PMC7521065; doi:10.15766/mep_2374-8265.10978)
Supplement: Supplementary file 1 — Sample Schedule.docxCase 1.docxCase 1 Handout for Residents.docxCase 1 Handout for Family.docxCase 1 Handout for Nurse.docxCase 1 Handout for Chaplain.docxCase 1 Handout for Social Worker.docxCase 1 Head CT Scan.docxCase 2.docxCase 2 Handout for Residents.docxCase 2 Handout for Family.docxCase 2 Handout for Nurse.docxCase 2 Handout for Chaplain.docxCase 2 Handout for Social Worker.docxCase 2 Head CT Scan.docxCase 2 Angiography.docxCase 2 SPECT Scan.docxChecklist.docxPre and Postsimulation Survey.docx [file mep_2374-8265.10978-s001.zip › K. Case 2 Handout for Family.docx]

## Case 2: Information for Family

Your husband, Thomas (Tommy), has been admitted to the hospital after a cardiac arrest caused him to fall off the roof. He had multiple broken ribs and other bones, and his heart stopped beating for a long time. The neurology team has done a great job of keeping you updated with what they were doing and finding, and you know the prognosis is grim.

You have never had a conversation with him about what his wishes were, if he were to have ended up in a situation like this, though you know he was an active, vital man, and he would have hated to see himself in this situation.

Unfortunately (in your mind), Tommy’s brother/sister, Jack/Jill, is going to be coming to the hospital today. You and your children have been in the hospital for Tommy’s whole hospital stay, and you are frustrated that Jack/Jill is only now coming in for a visit.

**Suggested questions to ask (wife):**

Is he in any pain?

Could I have prevented this?

What do all the monitors mean?

Can he hear me?

I’ve seen his leg moving around when they examine him. What does that mean?

**Suggested questions to ask (Jack/Jill):**

What else can you do for him?

People on TV come back from heart attacks all the time – why can’t Tommy?

Don’t people live in a coma for years?

My brother is a fighter – he’s always been lucky, and miracles have always happened to him – why not this time?
