# Supplementary material for: Brain Death Determination: An Interprofessional Simulation to Determine Brain Death and Communicate with Families Focused on Neurology Residents
Source: MedEdPORTAL. 2020 Sep 25;16:10978. doi: 10.15766/mep_2374-8265.10978 (PMC7521065; doi:10.15766/mep_2374-8265.10978)
Supplement: Supplementary file 1 — Sample Schedule.docxCase 1.docxCase 1 Handout for Residents.docxCase 1 Handout for Family.docxCase 1 Handout for Nurse.docxCase 1 Handout for Chaplain.docxCase 1 Handout for Social Worker.docxCase 1 Head CT Scan.docxCase 2.docxCase 2 Handout for Residents.docxCase 2 Handout for Family.docxCase 2 Handout for Nurse.docxCase 2 Handout for Chaplain.docxCase 2 Handout for Social Worker.docxCase 2 Head CT Scan.docxCase 2 Angiography.docxCase 2 SPECT Scan.docxChecklist.docxPre and Postsimulation Survey.docx [file mep_2374-8265.10978-s001.zip › L. Case 2 Handout for Nurse.docx]

## Case 2: Information for RN

Mr. O’Reilly is a 58-year-old man with no prior medical problems who was brought to the hospital ED 8 days ago after falling off a three-story roof onto the sidewalk. He had been complaining of chest pain that morning, though he ascribed it to a recent upper respiratory infection. After falling onto the sidewalk, a nurse who was out for a jog checked on him, and when she could not find a pulse, she started CPR. When EMS arrived 20 minutes later, he had not regained spontaneous circulation. Initial rhythm by EMS was non-shockable, and CPR continued for the 25 minutes until he arrived in the ED. There, he regained spontaneous circulation. A trauma CT revealed a basilar skull fracture, fractures of ribs 2-10 on the right, a right nondisplaced clavicle fracture, comminuted right humerus fractures, a right femoral neck fracture and a right tibial plateau fracture. A non-contrast head CT did not show intracranial hemorrhage. He was begun on a therapeutic hypothermia protocol, and the neurology consult service was called. When he was fully rewarmed, his exam remained poor, despite having been off sedation for 36 hours. He has remained off sedation for 4 days with no change in his exam.

You have been taking care of Mr. O’Reilly for the majority of his hospital stay, and you have gotten to know his wife and children very well. They are dealing with this situation really well, in your mind, and they very much understand what is going on. You have not met Jack/Jill (Tommy’s brother/sister), but you have heard Lisa (Tommy’s wife) talk about him/her, and you are worried that he/she will upset Lisa and her children. His exam, on your last neuro check was notable for: no pupil response in the left eye, and his right eye is swollen shut. He does not have a cough or gag. His right arm and leg are in surgical immobilizers. No movement in his left arm or leg.
