# Supplementary material for: Brain Death Determination: An Interprofessional Simulation to Determine Brain Death and Communicate with Families Focused on Neurology Residents
Source: MedEdPORTAL. 2020 Sep 25;16:10978. doi: 10.15766/mep_2374-8265.10978 (PMC7521065; doi:10.15766/mep_2374-8265.10978)
Supplement: Supplementary file 1 — Sample Schedule.docxCase 1.docxCase 1 Handout for Residents.docxCase 1 Handout for Family.docxCase 1 Handout for Nurse.docxCase 1 Handout for Chaplain.docxCase 1 Handout for Social Worker.docxCase 1 Head CT Scan.docxCase 2.docxCase 2 Handout for Residents.docxCase 2 Handout for Family.docxCase 2 Handout for Nurse.docxCase 2 Handout for Chaplain.docxCase 2 Handout for Social Worker.docxCase 2 Head CT Scan.docxCase 2 Angiography.docxCase 2 SPECT Scan.docxChecklist.docxPre and Postsimulation Survey.docx [file mep_2374-8265.10978-s001.zip › M. Case 2 Handout for Chaplain.docx]

## Case 2: Information for Chaplain

You have been called to see the family of Tommy O’Reilly, who is a 58-year-old man who has been admitted to the ICU after suffering a cardiac arrest that caused him to fall from the roof. His family is not particularly religious, though they asked for you to come spend time with them when things started to seem grim. Lisa (Tommy’s wife) is worried about the implications of Jack’s/Jill’s (Tommy’s brother/sister) arrival, as he/she is very religious, unlike Tommy and the rest of the family.
