# Supplementary material for: Brain Death Determination: An Interprofessional Simulation to Determine Brain Death and Communicate with Families Focused on Neurology Residents
Source: MedEdPORTAL. 2020 Sep 25;16:10978. doi: 10.15766/mep_2374-8265.10978 (PMC7521065; doi:10.15766/mep_2374-8265.10978)
Supplement: Supplementary file 1 — Sample Schedule.docxCase 1.docxCase 1 Handout for Residents.docxCase 1 Handout for Family.docxCase 1 Handout for Nurse.docxCase 1 Handout for Chaplain.docxCase 1 Handout for Social Worker.docxCase 1 Head CT Scan.docxCase 2.docxCase 2 Handout for Residents.docxCase 2 Handout for Family.docxCase 2 Handout for Nurse.docxCase 2 Handout for Chaplain.docxCase 2 Handout for Social Worker.docxCase 2 Head CT Scan.docxCase 2 Angiography.docxCase 2 SPECT Scan.docxChecklist.docxPre and Postsimulation Survey.docx [file mep_2374-8265.10978-s001.zip › N. Case 2 Handout for Social Worker.docx]

## Case 2: Information for Social Worker

You have been called to see the family of Tommy O’Reilly, who is a 58-year-old man who has been admitted to the ICU after suffering a cardiac arrest that caused him to fall from the roof. You have gotten to know Lisa (Tommy’s wife) and her children very well during the last few days. Lisa is holding up okay, as are the children, and they are expecting more bad news about Tommy’s condition.

You spoke with Jack/Jill, Tommy’s brother/sister, a couple days ago, at Lisa’s request, since she was having a tough time coping with his phone calls and questions. You are nervous that Jack will upset Lisa and the children.
