# Supplementary material for: Brain Death Determination: An Interprofessional Simulation to Determine Brain Death and Communicate with Families Focused on Neurology Residents
Source: MedEdPORTAL. 2020 Sep 25;16:10978. doi: 10.15766/mep_2374-8265.10978 (PMC7521065; doi:10.15766/mep_2374-8265.10978)
Supplement: Supplementary file 1 — Sample Schedule.docxCase 1.docxCase 1 Handout for Residents.docxCase 1 Handout for Family.docxCase 1 Handout for Nurse.docxCase 1 Handout for Chaplain.docxCase 1 Handout for Social Worker.docxCase 1 Head CT Scan.docxCase 2.docxCase 2 Handout for Residents.docxCase 2 Handout for Family.docxCase 2 Handout for Nurse.docxCase 2 Handout for Chaplain.docxCase 2 Handout for Social Worker.docxCase 2 Head CT Scan.docxCase 2 Angiography.docxCase 2 SPECT Scan.docxChecklist.docxPre and Postsimulation Survey.docx [file mep_2374-8265.10978-s001.zip › S. Pre and Postsimulation Survey.docx]

Please Affirm (Y) or Deny (N) the following statements:

1. I have participated in performing a brain death exam previously. Yes / No
2. I have previously discussed brain death with a patient’s family. Yes / No
3. Brain death is synonymous with death. Yes / No
4. I feel comfortable in discussing brain death with families. Yes / No
5. I know the prerequisites for performing the brain death exam. Yes / No
6. I know when ancillary testing should be performed to support the diagnosis of brain death. Yes / No
7. I feel comfortable performing the brain death exam. Yes / No
8. It should be a standard protocol to refer a neurologically injured patient to the to the Organ Procurement Agency before brain death testing. Yes / No
9. Physicians should be included in the discussion regarding organ donation. Yes / No
10. The discussion about organ donation should occur after brain death pronouncement. Yes / No
