# Supplementary material for: Brain Death Determination: An Interprofessional Simulation to Determine Brain Death and Communicate with Families Focused on Neurology Residents
Source: MedEdPORTAL. 2020 Sep 25;16:10978. doi: 10.15766/mep_2374-8265.10978 (PMC7521065; doi:10.15766/mep_2374-8265.10978)
Supplement: Supplementary file 1 — Sample Schedule.docxCase 1.docxCase 1 Handout for Residents.docxCase 1 Handout for Family.docxCase 1 Handout for Nurse.docxCase 1 Handout for Chaplain.docxCase 1 Handout for Social Worker.docxCase 1 Head CT Scan.docxCase 2.docxCase 2 Handout for Residents.docxCase 2 Handout for Family.docxCase 2 Handout for Nurse.docxCase 2 Handout for Chaplain.docxCase 2 Handout for Social Worker.docxCase 2 Head CT Scan.docxCase 2 Angiography.docxCase 2 SPECT Scan.docxChecklist.docxPre and Postsimulation Survey.docx [file mep_2374-8265.10978-s001.zip › _Educational_Summary_Report_10978.pdf]

MedEdPORTAL® The AAMC Journal of Teaching and Learning Resources

Original Publication Open Access

Brain Death Determination: An Interprofessional Simulation to Determine Brain Death and Communicate with Families Focused on Neurology Residents

Nicholas A. Morris, MD*, Eli E. Zimmerman, MD, Charles N. Pozner, MD, Galen V. Henderson, MD, Tracey A. Milligan, MD

*Corresponding Author: Nicholas.Morris@som.umaryland.edu

Abstract

Introduction: Significant variation exists in determining brain death despite an expectation of competence for all neurology residents. In addition, family discussions regarding brain death are challenging and may influence organ donation. **Methods:** We developed two simulations of increasing complexity for PGY 2 and PGY 3 neurology residents. High-fidelity mannequins were used to simulate patients; standardized actors portrayed family members. In the first simulation, residents determined brain death and shared this information with a grieving family. In the second simulation, residents determined brain death in a more complicated scenario, requiring ancillary testing and accurate result interpretation. Following the determination, residents met with a challenging family. The residents worked with an interdisciplinary team and responded to the family's emotions, used active listening skills, and supported the family through next steps. **Results:** Twelve residents completed the simulations. Prior to the simulation, three (25%) residents felt comfortable discussing a brain death diagnosis; following the simulation, eight (67%) residents felt comfortable/very comfortable discussing brain death. Prior to the simulation, eight (67%) residents stated they knew prerequisites for performing a brain death examination and seven (58%) agreed they knew indications for ancillary testing; these numbers increased to 100% following the simulation. The number of residents who felt comfortable performing the brain death exam increased from five (42%) to 10 (83%). **Discussion:** This simulation of determining brain death and leading difficult family meetings was well-received by neurology residents. Further work should focus on the effects of simulation-based education on practice variation and organ donation consent rates.

Keywords

Brain Death, Simulation, Communication, Nurse/Nurse Practitioner, Physician, Physician Assistant, Respiratory Therapist, Communication Skills, Critical Care Medicine, Neurology, Surgery, Neurosurgery, Standardized Patient

Educational Objectives

By the end of this activity, learners will be able to:

1. Accurately perform the coma exam for determination of brain death.
2. Identify indications for ancillary testing in the determination of brain death.
3. Correctly interpret ancillary testing in the determination of brain death.
4. Introduce the concept of brain death to family members prior to testing.
5. Properly communicate the diagnosis of brain death while avoiding misleading language (e.g., life support).
6. Describe specific protocols and state law regarding brain death.

Citation:
Morris NA, Zimmerman EE, Pozner CN, Henderson GV, Milligan TA. Brain death determination: an interprofessional simulation to determine brain death and communicate with families focused on neurology residents. *MedEdPORTAL*. 2020;16:10978.
https://doi.org/10.15766/mep_2374-8265.10978

Introduction

The determination of brain death is a high-stakes task that requires expertise. Residents have few experiences in determining brain death and are generally not performing the task independently, as most hospital policies require attending physicians to perform the examination.[1] The American Academy of Neurology first published guidelines for the determination of brain death in 1995, and updated these in 2010.[2] Despite clear guidelines, significant practice variation exists, some of which directly compromises patient care.[3,4] Such variation produces

Copyright © 2020 Morris et al. This is an open-access publication distributed under the terms of the Creative Commons Attribution license.

serious consequences for patients and fosters distrust in brain death determination by the public.[5]

All neurology residents are expected to be competent in accurately performing a brain death examination and diagnosing brain death by the completion of their residency.[6] Select institutions have developed brain death simulations in order to evaluate competency.[7,8] While these simulations have addressed the avoidance of pitfalls of brain death testing, they have not addressed the equally difficult challenge of discussing the diagnosis with grieving family members. A recent editorial on the topic, however, has called for simulations to include this pivotal aspect of the experience.[9] Indeed, studies have identified family understanding of brain death as a modifiable factor to increase organ donation consent rates.[10,11] As consent rates range from 35%-54% in the United States,[12] we are obliged to do anything we can to improve these numbers, especially as the demand for organ transplantation is rising while the number of brain dead donors is declining.[13,14] To this end, in 2012 we developed a simulation for neurology residents to practice brain death determination and family discussions thereof under the direct supervision of an expert in brain death with opportunities for debriefing with feedback. Brain death determination and associated family discussions typically involve multiple health professionals including physicians, nurses, respiratory therapists, social workers, and chaplains. We included these professionals in order to foster interprofessional communication, teamwork, and education. We believe this to be the first interprofessional simulation of brain death determination that incorporated a difficult family meeting. No other examples of brain death determinations simulation exist on *MedEdPORTAL*.

Methods

Development

Prior to the half-day simulation sessions, residents completed the following tasks: completed Cleveland Clinic's online education in Death by Neurologic criteria, reviewed a video of the coma exam, read an article on giving bad news,[15] attended or watched a video of a lecture on brain death that reviewed our hospital's protocols and those of the regional organ procurement agency, and attended a communication skills session. As some of these resources are no longer available, we recommend directing trainees to the Neurocritical Care Society's Brain Death Toolkit[16] for appropriate pretraining materials.

Equipment/Environment

- 1 simulated ICU room
- 1 meeting room
- SimMan 3G
- Ventilator
- Endotracheal tube with holder
- Gown
- IV pump and line
- Monitor
- Butterfly needles
- 60 cc catheter tip syringe
- Emesis basin
- Absorbable underpads
- Q tips
- Suction catheter with O2 tubing
- Cup of ice water
- Artificial blood concentrate
- Transparent film dressing
- Gelefects and makeup to mimic orbital ecchymosis
- Miami-J cervical collar
- Checklist for determination and declaration of brain death
- Paper handout of CT scan with large intracerebral hemorrhage
- Paper handout of CT showing diffuse cerebral edema
- Paper handout of angiogram still showing carotid injection with extracranial but no intracranial flow
- Paper handout of HMPAO-SPECT with "empty lightbulb" sign
- Separate paper handouts with case information for each case for residents, family, nurse, chaplain, and social worker

Personnel

The target learners were first- and second-year neurology residents who performed brain death determination and led family discussions as a team. The other team members included: a respiratory therapist who assisted in ventilator management during apnea testing; an intensive care unit nurse who assisted with brain death determination and apnea testing and took part in family discussion; a social worker and chaplain who took part in the family discussion; and two actors portraying family members for each scenario who took part in family discussions.

Implementation

On the day of the simulation, the two residents arrived at 8 a.m. to the Neil and Elise Wallace STRATUS Center for Medical Simulation at Brigham and Women's Hospital. They were provided a 20-minute introduction followed by a pretest. Residents then completed two simulations of increasing difficulty. A typical schedule is provided (Appendix A).

We allotted 70 minutes for the first simulation (Appendix B); 20 minutes for performing the brain death exam (including apnea testing), 10 minutes to huddle with the interprofessional team,

Copyright © 2020 Morris et al. This is an open-access publication distributed under the terms of the Creative Commons Attribution license.

20 minutes to conduct a family meeting, and 20 minutes for debriefing. Residents were provided a written case summary that placed the interaction in context. They were provided general guidance for the family meeting (Appendix C). Prior to the simulation, the residents were informed that they had already conducted an initial family meeting where the concept of brain death had been introduced and that one of two brain death examinations had already been completed with the attending neurologist on rounds (our institutional brain death policy requires two brain death exams with one of the exams to be completed by the attending physician; the apnea testing only need be completed once). We instructed the residents to complete brain death testing, huddle with the interprofessional team, and then discuss the results with the family.

The scenario began with residents entering the simulated ICU room where a nurse and respiratory therapist who had been provided background of the case were already positioned. Successful completion of the task required the residents to rule out confounders to the exam. In this case, they had to recognize that the patient was hypothermic, and then warm the patient prior to completing the exam. The resident also had to review the imaging to ensure that they understood the cause of the irreversible coma as explained by the radiographic findings. The residents then performed and documented a coma examination. They were instructed to identify and document the absence of responsiveness, cranial nerve reflexes, and motor activity. After ensuring that the patient was hemodynamically stable and that arterial blood gas values were within an acceptable range, they preoxygenated the patient, provided oxygen via cannula inside the endotracheal tube to the level of the carina at 6L/min. They then disconnected the ventilator and observed closely for spontaneous respirations. When absent, they had to perform an arterial blood gas after 10 minutes. Once the blood gas was obtained, they had to reconnect the patient to the ventilator. Finally, they had to correctly interpret the arterial blood gas as to be consistent with brain death and appropriately document their findings.

The residents next huddled with the interprofessional team to discuss their approach to the family meeting. Actors, who portrayed the family members, were each provided a script (Appendix D) with the appropriate context and suggested questions to ask the residents. The interprofessional team included the nurse, as well as a social worker from the hospital's neurology unit, and a hospital chaplain who each played their own roles. Each team member was provided a unique script with pertinent information to share with the rest of the team to facilitate the family discussion (Appendices E, F, G). The team then met with the family to communicate the diagnosis, answer questions, and provide support. Following the family meeting, an expert in brain death and brain death communication guided a debriefing. Imaging for the case is provided (Appendix H).

After a 15-minute break, the residents began a second case (guided by Appendix I). They were provided a written case summary that described the background of the case (Appendix J). In this case the residents consulted on a patient who had a traumatic cardiac arrest. For this case, the residents were instructed to huddle with the interprofessional team, have a meeting to introduce the concept of brain death to the family, perform the brain death exam including apnea testing, rehuddle with the team, and then meet again with the family to discuss the results of the test and the next steps. We provided the residents with general guidelines for both the pre- and postexam family meetings. Again, all participants were given guiding scripts to guide the family meeting (Appendices K, L, M, N). Imaging for the case is provided (Appendices 0, P, Q).

In order to successfully complete the brain death determination, residents had to recognize the need for ancillary testing due to an inability to complete the exam because of periorbital edema that prevented eyelid retraction, as well as hemotympanum precluding cold caloric testing. In addition, the patient's oxygen saturation fell below 85% for a sustained period during apnea testing, which required aborting this procedure. If they chose to repeat the exam using noninvasive ventilation, the patient once again desaturated. We provided ancillary testing results upon request, including conventional angiography and nuclear studies. Our institution's checklist for determination and declaration of brain death (Appendix R) was also provided. Residents then had to appropriately complete documentation.

Following the brain death determination, the residents huddled again with the interprofessional team and then returned to meet with the family and disclose the results. In order to challenge the residents, the preexam meeting only included the patient's wife, but the postexam meeting included a sibling who had just arrived from out of town. We instructed the actors portraying the wife and sibling that the patient's wishes were unknown. We suggested they ask loaded questions such as, "Is he in pain?," "Can he hear me?," and, "Couldn't he be saved by a miracle?" The sibling, who was portrayed as highly religious, challenged the diagnosis of brain death and suggested that we "leave it up to God." Successful completion of the meeting required that the residents use simple words to explain the clinical scenario, avoid medical jargon, incorporate visual aids, provide the time of death

Copyright © 2020 Morris et al. This is an open-access publication distributed under the terms of the Creative Commons Attribution license.

and use the word “dead,” check for comprehension, use active listening, avoid suggestions of hope, and explain next decision-making steps without mention of organ donation.

Assessment

We gave the residents a brief 10-question pre- and postsimulation survey (Appendix S) to assess their experience with and understanding of brain death determination.

Debriefing

Following the simulation, an expert in brain death determination debriefed all participants, loosely following the principles of the debriefing with good judgment model.[17] Suggested topics for the debriefing included:

1. Appropriate ordering of ancillary tests.
2. Preparation for the family meeting.
3. Use of specific language to clarify and/or not confuse the situation.
4. Incorporation of other members of the team into the family meeting.

Results

Twelve first- and second-year residents completed the simulations. Prior to the simulations, nine (75%) had performed a brain death exam and 10 (83%) had discussed brain death with a patient’s family. Prior to the simulation, three (25%) residents felt comfortable discussing the diagnosis of brain death; following the simulation eight (67%) residents felt comfortable or very comfortable discussing the diagnosis of brain death. Only one remained uncomfortable discussing the diagnosis of brain death. Prior to the simulation, two of the participants did not consider brain death synonymous with death and following the simulation all residents agreed brain death is synonymous with death. Prior to the simulation, eight (67%) residents stated they knew the prerequisites for performing a brain death examination and seven (58%) agreed that they knew when ancillary testing should be used. These numbers increased to 100% following the simulation. The number of residents who felt comfortable performing the brain death exam increased from five (42%) prior to the simulation to 10 (83%) following the simulation. Four residents completed additional course feedback surveys, and all described the sessions as inspiring and novel.

Discussion

We successfully implemented a two-part simulation on brain death determination. Residents gained experience in the brain death examination, including how to rule out common confounders and the proper use of ancillary testing. Working with an interprofessional team, they also practiced family meetings during which they introduced the concept of brain death while managing family emotions. Postscenario debriefing enabled faculty to address both strengths and weaknesses in cognitive, psychomotor, and affective domains. Mannequin-based simulation is well-suited to brain death determination scenarios, as the mannequin need not be capable of mimicking a functional nervous system. Incorporation of standardized actors to recreate family meetings allowed residents to explore emotions using a team-based approach. Participants found the scenarios realistic and valuable.

There were numerous benefits of this simulation. Foremost, junior residents acquired hands-on experience with the performance of a coma exam in a low-stakes situation. They were able to receive additional supervision from senior residents and dedicated faculty members. Senior residents were able to lead simulated family meetings, work with an interdisciplinary team, and navigate a challenging patient situation. It is unusual for residents to have their conversations with families be observed, let alone receive feedback for them. Residents were also able to review the core educational content of the determination of brain death and the need for ancillary testing.

We discovered several limitations to our simulation project. Foremost, the simulations were time and resource intensive. To optimize the experience, we incorporated an interprofessional team including neurology residents, intensive care unit nurses, respiratory therapists, social workers, chaplains, as well as neurology attending physicians experienced in brain death determination and simulation operations specialists. Coordinating schedules and sacrificing so many participants from clinical duties for 4 hours per simulation session was challenging, but we felt worth the investment. Second, we were limited in our evaluation of outcomes. We did not follow the residents over time to ascertain if our intervention resulted in improvement in performance in the clinical setting. While our institution does utilize a checklist for determination and declaration of brain death (Appendix R), the checklist could be expanded to include critical actions that do not include the minimum diagnostic criteria. Such critical actions might include uncovering the patient to observe respirations and preoxygenating the patient prior to apnea testing. Future work should include more rigorous assessment of performance between scenarios as well as the durability of any improvement in skills. Assessment of observed clinical performance pre- and postintervention would also be valuable. We could even implement the updated checklist into real brain death determinations by the attending physician supervising

Copyright © 2020 Morris et al. This is an open-access publication distributed under the terms of the Creative Commons Attribution license.

the resident. We were also unable to evaluate the effect of our family meeting simulations on residents' performances in actual family meetings. Future work could consider again using checklist evaluations during real family meetings. Alternatively, we could survey families to receive direct feedback on resident performance.

Recently, several prominent lawsuits have questioned the legitimacy of brain death determination.[5] The American Academy of Neurology's response has been to advocate for education initiatives and promote brain death training and credentialing programs to ensure that brain death determinations are made according to established guidelines.[18] Sadly, studies persistently show that physicians fail to execute guideline-based brain death determinations. Given the stakes of the exam, this failure is appalling. More training is required. We should consider mannequin-based simulation for credentialing so that candidates may demonstrate appropriate behaviors, not simply rote knowledge. To do so, more work is required to validate brain death simulations and assessment tools for high stakes examination. Large-scale efforts to improve uniformity of brain death determination should be monitored for their effects on public perception of the diagnosis. Furthermore, efforts to improve family meetings and family understanding should be tracked to assess their impact on organ donation consent rates. Brain death determination is one of the most serious skills that a physician performs, both in the mechanics of the examination and in the communication skills required to discuss with a patient's family.

Appendices

A. Sample Schedule.docx
B. Case 1.docx
C. Case 1 Handout for Residents.docx
D. Case 1 Handout for Family.docx
E. Case 1 Handout for Nurse.docx
F. Case 1 Handout for Chaplain.docx
G. Case 1 Handout for Social Worker.docx
H. Case 1 Head CT Scan.docx
I. Case 2.docx
J. Case 2 Handout for Residents.docx
K. Case 2 Handout for Family.docx
L. Case 2 Handout for Nurse.docx
M. Case 2 Handout for Chaplain.docx
N. Case 2 Handout for Social Worker.docx
O. Case 2 Head CT Scan.docx
P. Case 2 Angiography.docx
Q. Case 2 SPECT Scan.docx
R. Checklist.docx
S. Pre and Postsimulation Survey.docx

All appendices are peer reviewed as integral parts of the Original Publication.

Nicholas A. Morris, MD: Assistant Professor, Department of Neurology, Program in Trauma, University of Maryland School of Medicine; ORCID: https://orcid.org/0000-0002-1270-9805

Eli E. Zimmerman, MD: Assistant Professor, Department of Neurology, Vanderbilt University School of Medicine

Charles N. Pozner, MD: Associate Professor, Department of Emergency Medicine, Brigham and Women's Hospital, Harvard Medical School

Galen V. Henderson, MD: Assistant Professor, Department of Neurology, Brigham and Women's Hospital, Harvard Medical School

Tracey A. Milligan, MD: Assistant Professor, Department of Neurology, Brigham and Women's Hospital, Harvard Medical School

Disclosures
None to report.

Funding/Support
None to report.

Prior Presentations
Morris N, Zimmerman E, Henderson G, Milligan T. Death by neurologic criteria and difficulty family discussions: a resident simulation experience. Poster presented at: Annual Meeting of the American Neurological Association; October 12-14, 2014; New Orleans, LA.

Ethical Approval
Reported as not applicable.

References

1. Greer DM, Wang HH, Robinson JD, Varelas PN, Henderson GV, Wijdicks EFM. Variability of brain death policies in the United States. *JAMA Neurol*. 2016;73(2):213-218. https://doi.org/10.1001/jamaneurol.2015.3943
2. Wijdicks EFM, Varelas PN, Gronseth GS, Greer DM. Evidence-based guideline update: determining brain death in adults: report of the Quality Standards Subcommittee of the American Academy of Neurology. *Neurology*. 2010;74(23): 1911-1918. https://doi.org/10.1212/WNL.0b013e3181e242a8

Copyright © 2020 Morris et al. This is an open-access publication distributed under the terms of the Creative Commons Attribution license.

3. Shappell CN, Frank JI, Husari K, Sanchez M, Goldenberg F, Ardelt A. Practice variability in brain death determination: a call to action. *Neurology*. 2013;81(23):2009-2014. https://doi.org/10.1212/01.wnl.0000436938.70528.4a

4. Braksick SA, Robinson CP, Gronseth GS, Hocker S, Wijdicks EFM, Rabinstein AA. Variability in reported physician practices for brain death determination. *Neurology*. 2019;92(9):e888-e894.

5. Lewis A, Bernat JL, Blosser S, et al. An interdisciplinary response to contemporary concerns about brain death determination. *Neurology*. 2018;90(9):423-426. https://doi.org/10.1212/WNL.0000000000005033

6. Lewis SL, Józefowicz RF, Kilgore S, Dhand A, Edgar L. Introducing the neurology milestones. *J Grad Med Educ*. 2014;6(1 Suppl 1):102-104. https://doi.org/10.4300/JGME-06-01s1-34

7. MacDougall BJ, Robinson JD, Kappus L, Sudikoff SN, Greer DM. Simulation-based training in brain death determination. *Neurocrit Care*. 2014;21(3):383-391. https://doi.org/10.1007/s12028-014-9975-x

8. Hocker S, Schumacher D, Mandrekar J, Wijdicks EFM. Testing confounders in brain death determination: a new simulation model. *Neurocrit Care*. 2015;23(3):401-408. https://doi.org/10.1007/s12028-015-0130-0

9. Bernat JL, Brust JCM. Strategies to improve uniformity in brain death determination. *Neurology*. 2019;92(9):401-402.

10. Siminoff LA, Gordon N, Hewlett J, Arnold RM. Factors influencing families' consent for donation of solid organs for transplantation. *JAMA*. 2001;286(1):71-77. https://doi.org/10.1001/jama.286.1.71

11. Gortmaker SL, Beasley CL, Sheehy E, et al. Improving the request process to increase family consent for organ donation. *J Transpl Coord*. 1998;8(4):210-217. https://doi.org/10.1177/090591999800800404

12. Sheehy E, Conrad SL, Brigham LE, et al. Estimating the number of potential organ donors in the United States. *N Engl J Med*. 2003;349(7):667-674. https://doi.org/10.1056/NEJMsa021271

13. Klein AS, Messersmith EE, Ratner LE, Kochik R, Baliga PK, Ojo AO. Organ donation and utilization in the United States, 1999-2008. *Am J Transplant*. 2010;10(4 Pt 2):973-986. https://doi.org/10.1111/j.1600-6143.2009.03008.x

14. Kompanje EJO, de Groot YJ, Bakker J. Is organ donation from brain dead donors reaching an inescapable and desirable nadir? *Transplantation*. 2011;91(11):1177-1180. https://doi.org/10.1097/TP.0b013e3182180567

15. Baile WF, Buckman R, Lenzi R, Glober G, Beale EA, Kudelka AP. SPIKES—a six-step protocol for delivering bad news: application to the patient with cancer. *Oncologist*. 2000;5(4):302-311. https://doi.org/10.1634/theoncologist.5-4-302

16. Digital Education: Brain Death Toolkit. Neurocritical Care Society. Accessed August 19, 2020. https://www.neurocriticalcare.org/education/digital-education/brain-death-toolkit

17. Rudolph JW, Simon R, Rivard P, Dufresne RL, Raemer DB. Debriefing with good judgment: combining rigorous feedback with genuine inquiry. *Anesthesiol Clin*. 2007;25(2):361-376. https://doi.org/10.1016/j.anclin.2007.03.007

18. Lewis A, Bernat JL, Blosser S, et al. An interdisciplinary response to contemporary concerns about brain death determination. *Neurology*. 2018;90(9):423-426. https://doi.org/10.1212/WNL.0000000000005033

Received: September 10, 2019
Accepted: March 30, 2020
Published: September 25, 2020

Copyright © 2020 Morris et al. This is an open-access publication distributed under the terms of the Creative Commons Attribution license.
