# Supplementary figures and images for: Brain Death Determination: An Interprofessional Simulation to Determine Brain Death and Communicate with Families Focused on Neurology Residents
Source: MedEdPORTAL. 2020 Sep 25;16:10978. doi: 10.15766/mep_2374-8265.10978 (PMC7521065; doi:10.15766/mep_2374-8265.10978)

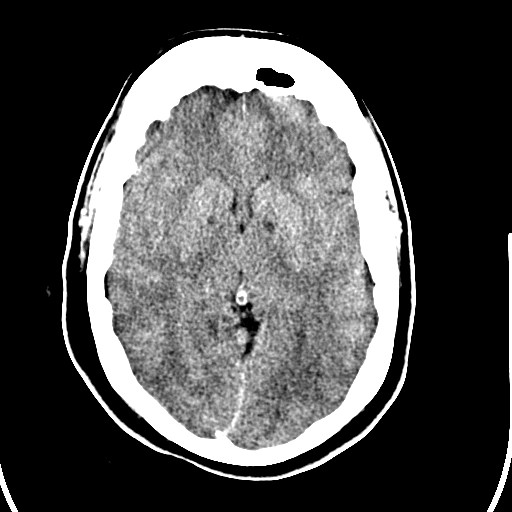


Author owned image.

Supplement: Supplementary file 1 — Sample Schedule.docxCase 1.docxCase 1 Handout for Residents.docxCase 1 Handout for Family.docxCase 1 Handout for Nurse.docxCase 1 Handout for Chaplain.docxCase 1 Handout for Social Worker.docxCase 1 Head CT Scan.docxCase 2.docxCase 2 Handout for Residents.docxCase 2 Handout for Family.docxCase 2 Handout for Nurse.docxCase 2 Handout for Chaplain.docxCase 2 Handout for Social Worker.docxCase 2 Head CT Scan.docxCase 2 Angiography.docxCase 2 SPECT Scan.docxChecklist.docxPre and Postsimulation Survey.docx [file mep_2374-8265.10978-s001.zip › O. Case 2 Head CT Scan.docx]

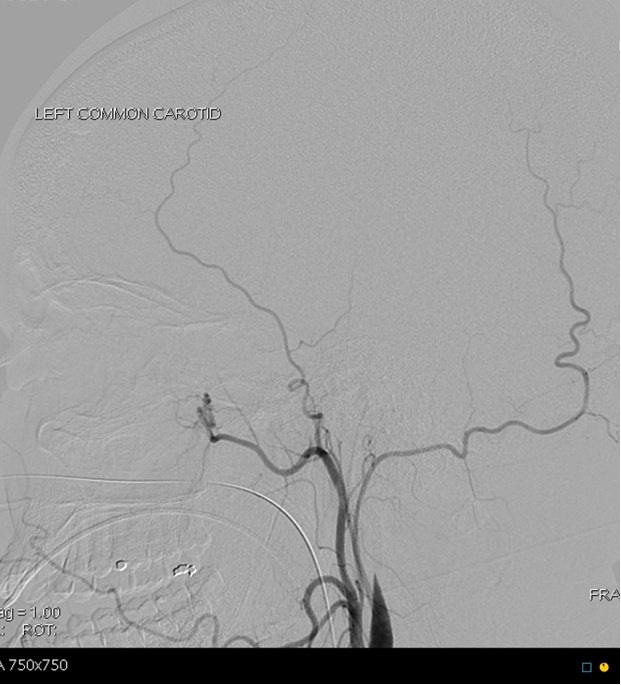


Author owned image.

Supplement: Supplementary file 1 — Sample Schedule.docxCase 1.docxCase 1 Handout for Residents.docxCase 1 Handout for Family.docxCase 1 Handout for Nurse.docxCase 1 Handout for Chaplain.docxCase 1 Handout for Social Worker.docxCase 1 Head CT Scan.docxCase 2.docxCase 2 Handout for Residents.docxCase 2 Handout for Family.docxCase 2 Handout for Nurse.docxCase 2 Handout for Chaplain.docxCase 2 Handout for Social Worker.docxCase 2 Head CT Scan.docxCase 2 Angiography.docxCase 2 SPECT Scan.docxChecklist.docxPre and Postsimulation Survey.docx [file mep_2374-8265.10978-s001.zip › P. Case 2 Angiography.docx]

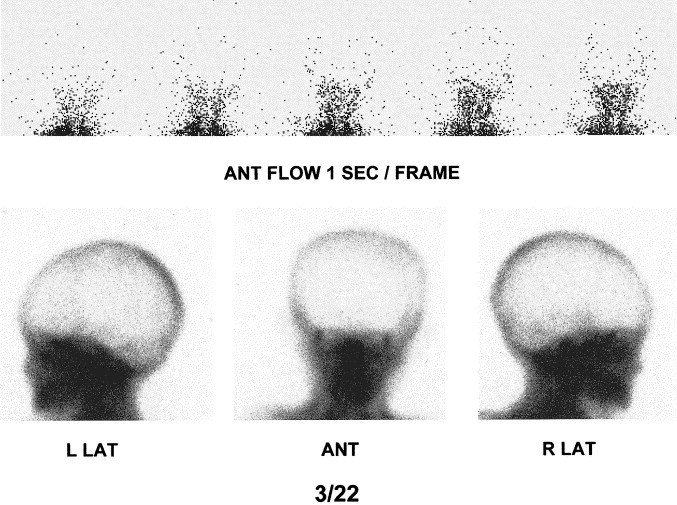


Author owned image.

Supplement: Supplementary file 1 — Sample Schedule.docxCase 1.docxCase 1 Handout for Residents.docxCase 1 Handout for Family.docxCase 1 Handout for Nurse.docxCase 1 Handout for Chaplain.docxCase 1 Handout for Social Worker.docxCase 1 Head CT Scan.docxCase 2.docxCase 2 Handout for Residents.docxCase 2 Handout for Family.docxCase 2 Handout for Nurse.docxCase 2 Handout for Chaplain.docxCase 2 Handout for Social Worker.docxCase 2 Head CT Scan.docxCase 2 Angiography.docxCase 2 SPECT Scan.docxChecklist.docxPre and Postsimulation Survey.docx [file mep_2374-8265.10978-s001.zip › Q. Case 2 SPECT Scan.docx]

#
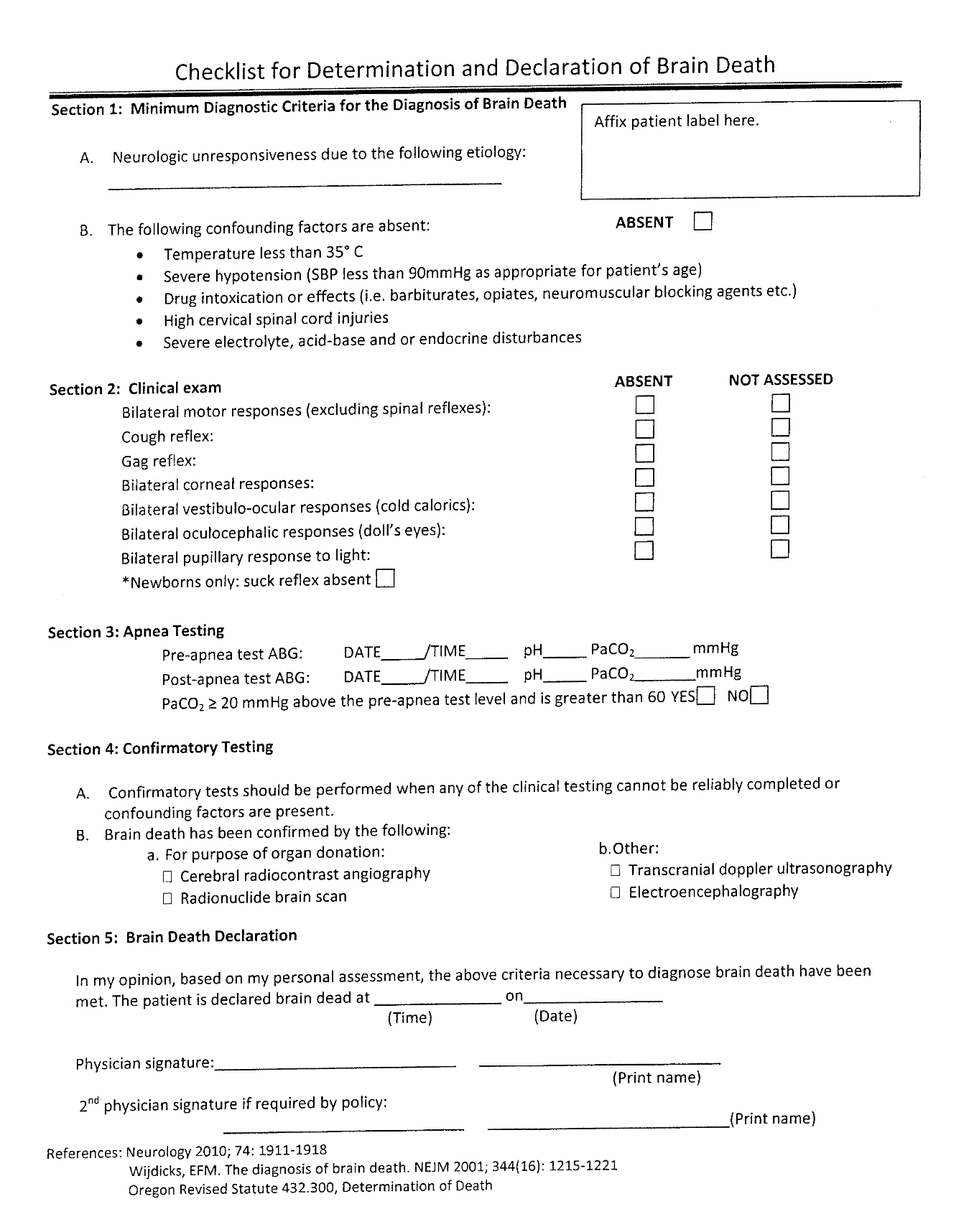

Supplement: Supplementary file 1 — Sample Schedule.docxCase 1.docxCase 1 Handout for Residents.docxCase 1 Handout for Family.docxCase 1 Handout for Nurse.docxCase 1 Handout for Chaplain.docxCase 1 Handout for Social Worker.docxCase 1 Head CT Scan.docxCase 2.docxCase 2 Handout for Residents.docxCase 2 Handout for Family.docxCase 2 Handout for Nurse.docxCase 2 Handout for Chaplain.docxCase 2 Handout for Social Worker.docxCase 2 Head CT Scan.docxCase 2 Angiography.docxCase 2 SPECT Scan.docxChecklist.docxPre and Postsimulation Survey.docx [file mep_2374-8265.10978-s001.zip › R. Checklist.docx]
